# Serum matrix metalloproteinase-7 for discriminating biliary atresia: a diagnostic accuracy and validation study

**DOI:** 10.1186/s12967-024-05442-x

**Published:** 2024-07-08

**Authors:** Jingying Jiang, Rui Dong, Min Du, Gong Chen, Jingyun Yang, Xinbao Xie, Yifan Yang, Weili Yan, Shan Zheng

**Affiliations:** 1 Department of Pediatric Surgery, Shanghai Key Laboratory of Birth Defect, Key Laboratory of Neonatal Disease, Children’s Hospital of Fudan University, Ministry of Health, 399 Wan Yuan Road, Shanghai, 201102 China; 2 Rush Alzheimer’s Disease Center, Rush University Medical Center, Chicago, IL USA; 3 Department of Neurological Sciences, Rush University Medical Center, Chicago, IL USA; 4 Department of Hepatology, Children’s Hospital of Fudan University, 399 Wan Yuan Road, Shanghai, 201102 China; 5 Department of Clinical Epidemiology, Clinical Trial Unit, Children’s Hospital of Fudan University, 399 Wan Yuan Road, Shanghai, 201102 China

**Keywords:** Cholestasis, Biliary atresia, Diagnostic accuracy, Neonate, Cutoff value, Biomarker, MMP-7

## Abstract

**Background:**

Prompt and precise differential diagnosis of biliary atresia (BA) among cholestatic patients is of great importance. Matrix metalloproteinase-7 (MMP-7) holds great promise as a diagnostic marker for BA. This study aimed to investigate the accuracy of age-specific serum MMP-7 for discriminating BA from other cholestatic pediatric patients.

**Methods:**

This was a single center diagnostic accuracy and validation study including both retrospective and prospective cohorts. Serum MMP-7 concentrations were measured using an ELISA kit, the trajectory of which with age was investigated in a healthy infants cohort aged 0 to 365 days without hepatobiliary diseases (*n* = 284). Clinical BA diagnosis was based on intraoperative cholangiography and subsequent histological examinations. The diagnostic accuracy of age-specific cutoffs of serum MMP-7 were assessed in a retrospective cohort of cholestatic patients (*n* = 318, with 172 BA) and validated in a prospective cohort (*n* = 687, including 395 BA).

**Results:**

The MMP-7 concentration declines non-linearly with age, showing higher levels in healthy neonates as well as higher cutoff value in neonatal cholestasis. The area under the ROC curve (AUROC) was 0.967 (95% confidence interval [CI]: 0.946–0.988) for the retrospective cohort, and the cutoff of 18 ng/mL yielded 93.0% (95%CI: 88.1-96.3%), 93.8% (95%CI: 88.6-97.1%), 94.7% (95%CI: 90.1-97.5%), and 91.9% (95%CI: 86.4-95.8%) for sensitivity, specificity, positive predictive value (PPV), and negative predictive value (NPV), respectively. The performance of MMP-7 was successfully validated in the larger prospective cohort, resulting in a diagnostic sensitivity of 95.9% (379/395; 95% CI: 93.5–97.7%), a specificity of 87.3% (255/292; 95% CI: 83.0–90.9%), a PPV of 91.1% (379/416; 95% CI: 87.9–93.7%), and a NPV of 94.1% (255/271; 95% CI: 90.6–96.6%), respectively. Besides, higher cutoff value of 28.1 ng/mL achieved the best sensitivity, specificity, PPV, and NPV for infants aged 0–30 days, which was 86.4% (95% CI: 75.0–94.0%), 95.5% (95% CI: 77.2–99.9%), 98.1% (95% CI: 89.7–100%), and 72.4% (95% CI: 52.8–87.3%), respectively.

**Conclusions:**

The serum MMP-7 is accurate and reliable in differentiating BA from non-BA cholestasis, showing its potential application in the diagnostic algorithm for BA and significant role in the future research regarding pathogenesis of BA.

**Supplementary Information:**

The online version contains supplementary material available at 10.1186/s12967-024-05442-x.

## Introduction

Biliary atresia (BA) is a severe liver disease characterized by fibro-inflammatory destruction of bile ducts that affects 1/8000–10,000 neonates and infants. Since 1959, Kasai portoenterostomy (KPE) has been the standard treatment strategy to restore bile flow [1–4]. While the prognosis following KPE is indeterminate, the age at which infants with BA undergo KPE is considered a positive prognostic factor, as those who receive surgery before 60 days after birth generally achieve better outcomes [3, 5–7]. However, prompt treatment relies on early and precise diagnosis. To date, it remains difficult to achieve perfect accuracy with either liver function assays or grayscale ultrasound scans, which are the most widely used tests in clinical practice [8–10]. In 2018, Dong et al. established a nomogram diagnostic model, slightly improving the diagnostic accuracy [8].

Matrix metalloproteinase-7 (MMP-7) plays an important role in remodeling the extracellular matrix (ECM), which is closely related to liver damage and liver fibrosis progression [11–13]. Compared with other cholestatic diseases, biliary atresia always shows higher and more rapid progression of liver fibrosis. It was noticed that MMP-7 levels were higher in BA patients especially those with higher stages of liver fibrosis [12], indicating MMP-7 might be involved in the pathogenesis and progression of BA. In 2018, Yang et al. reported the high diagnostic accuracy of MMP-7 in BA with the AUC of 0.99 [14]. Recently, several studies have further confirmed the clinical value of serum MMP-7 levels in discriminating BA from other patients, most of which were based on ELISA assays, however, showing large variation in cutoff values [14–24]. Besides, the study by Chi et al. revealed a dynamic increasing trend of the serum MMP-7 levels in a cohort of BA infant patients after Kasai surgery [18]. However, little is known about the natural distribution and dynamic trend of serum MMP-7 levels with age across the diagnosing window of BA in normal infant population who were not affected by cholestatic diseases, which is essential for identifying optimal clinical discriminative cutoff. Also, to date, a well-designed diagnostic study with prospective validation is lacking, limiting the application of MMP-7 in clinical practice.

Thus, we conducted this study to illustrate the centile distribution of serum MMP-7 for age in a non-affected cohort of neonates and infants with similar age with BA, based on which to establish age-specific serum MMP-7 cutoff values for diagnosing BA and validate these cutoffs in a prospective cohort.

## Methods

### Study design

**Fig. 1:**
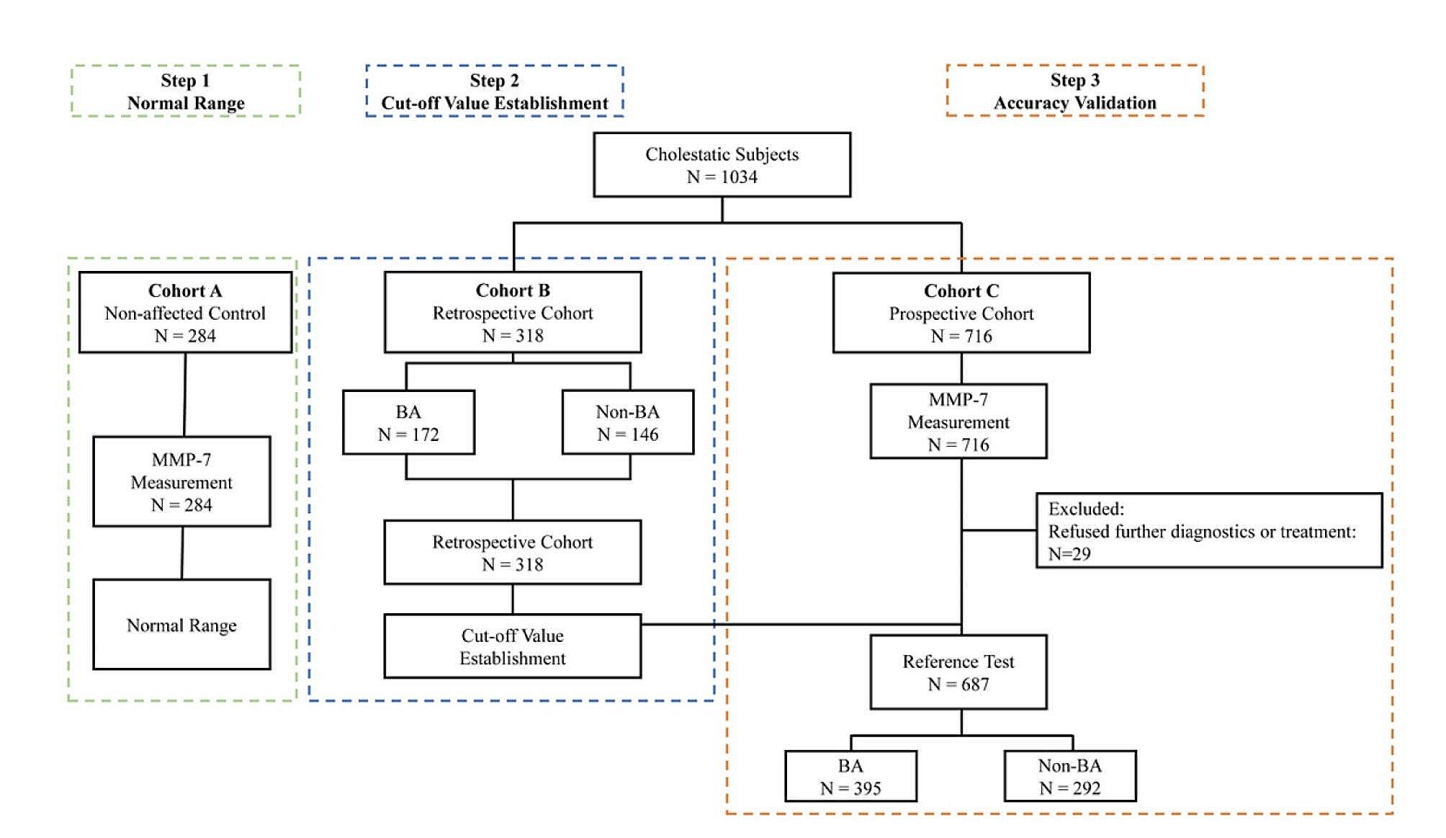
Flow chart of the study patients and design

This was a single-center diagnostic accuracy study and a validation study. Three cohorts of infants were included. We established the centile distribution trend of serum MMP-7 for age in infants without any hepatobiliary diseases (cohort A, *n* = 284), and then identified a cut-off value to differentiate BA from non-BA cholestatic patients from a retrospective cohort (cohort B, *n* = 318) and further validated its accuracy with a prospectively recruited patient cohort (cohort C, *n* = 687) (Fig. 1).

This work was performed in compliance with the Declaration of Helsinki and other relevant regulations. The research protocol was submitted for consideration, comment, guidance and approval to the ethics committee before the study begins. Informed consent was obtained from the guardian of each participant before enrollment, otherwise, the subject would be excluded from final analysis. Patients’ names, initials, dates of birth or other personal or identifying information were confidential and not used.

### Setting and study subjects

This study was conducted at an urban tertiary care academic children’s hospital in Shanghai, China, one of the largest medical centers for BA both home and abroad, where over 70% of BA patients (over 200 per year) were from all over the country.

Cohort A: Infants younger than 1 year of age who were admitted to the hospital for reasons other than hepatobiliary diseases or jaundice between July 2020 and March 2022 were screened for eligibility and enrolled as non-affected controls. Medical records, including biochemistry tests and final diagnosis, were reviewed before enrollment.

Cohort B and C: Infants who consulted for obstructive jaundice with a serum direct bilirubin level > 17 µmol/L and accounting for > 20% of total bilirubin were consecutively enrolled and defined as cholestatic subjects, for whom biliary atresia should be suspected and excluded or confirmed as soon as possible during clinical practice. These cases consisted of a retrospective cohort (from October 2018 to December 2019, Cohort B) and a prospective cohort (from June 2020 to March 2022, Cohort C).

### Index test

#### Measuring serum MMP-7 concentrations by ELISA

On the day of admission of each patient of the three cohorts, the serum samples of 500ul were obtained for MMP-7 measurement and stored at -80^o^C before measurement. Serum MMP-7 concentrations were measured using an ELISA kit (R&D Systems, DMP700, Minneapolis, MN, USA) according to the manufacturer’s protocol, based on the 20-fold diluted serum samples. Every measurement would include standards and controls. A standard curve was generated based on a log/log curve-fit for each measurement. All measurements were performed by WuXi Diagnostics (Shanghai, China), who was blinded to other test results. Each sample was provided with three technical replicates, and the mean was recorded.

#### Reference standard

Baseline assessments, including demographic characteristics, medical histories, and biochemistry tests, were performed for all patients. The liver function tests including total bilirubin (TB), direct bilirubin (DB), gamma-glutamyl transferase (GGT), aspartate aminotransferase (AST), alanine aminotransferase (ALT), alkaline phosphatase (ALP), and total bile acid (TBA) were examined in the hospital lab center according to uniform standard protocols.

BA diagnoses were made based on intraoperative cholangiography and subsequent histological examinations of liver biopsies [1]. Non-BA patients were confirmed by intraoperative cholangiography showing a patent biliary tree, and/or percutaneous transhepatic biopsy excluding BA, and/or genetic tests showing certain genetic mutations, and/or alleviation of symptoms without surgical intervention during follow-up for at least 3 months.

The histological features of all liver biopsy specimens were measured and recorded by the same pathologist (YYM). The grades of inflammation and stages of fibrosis were defined according to the Batts-Ludwig scale system, ranging from G1 to G4 and S0 to S4.

### Sample size planning

To describe the centile distribution of serum MMP-7 concentrations for age (days), a cross-sectional cohort of 120 non-affected infants without hepatobiliary diseases was required according to the CLSI C28-A3 guideline [25]. To identify a cutoff value of serum MMP-7 to achieve the best diagnostic accuracy in discriminating BA from non-BA patients, which was hypothesized a sensitivity of over 90% and a specificity of over 90% though previous studies showed higher accuracy [15–18], with a two-tailed α of 0.05 and Δ of 0.05, a total of 304 cholestatic participants including 152 BA patients (50%) would be sufficient to achieve a power of 0.8 for the developmental study. For the prospective validation, we planned a larger sample size. We assumed a screening failure rate of 10% with the consideration of unclear diagnosis. However, based on our experience, the screening failure rate was much lower than 10%, so the calculated sample size would be far more than sufficient.

### Statistical analysis

Demographic and clinical characteristics of patients were summarized using conventional descriptive statistics, n (%) for categorical variables and median and quartiles (Q1, Q3) for continuous variables.

The centile distribution of MMP-7 for age was derived utilizing the Lambda-Mu-Sigma (LMS) method in the overall cohort and by age groups. Since the data did not follow a normal distribution using the Shapiro-Wilk test, nonparametric tests were performed including Chi-squared and Mann–Whitney U tests, and *P* values were based on Bonferroni adjustment. Kruskal-Wallis tests were applied for multiple comparisons. The reference range was determined using a nonparametric method (2.5th − 97.5th percentiles) according to the CLSI C28-A3 guideline [25]. Receiver operating characteristic (ROC) curves were conducted with clinically confirmed BA as a dichotomous outcome variable and MMP-7 level as the test variable, and the area under the curve (AUC) and 95% confidence intervals (CIs) were reported as a measure of accuracy. We used the maximum value of Youden’s index as a criterion for selecting the optimal cut-off point. When the maximum value of Youden’s index was achieved for multiple specificity values, the optimal cutoffs were defined based on achieving the maximum sensitivity. The sensitivity, specificity, positive predictive value (PPV), and negative predictive value (NPV) and their 95% CIs were also reported to indicate diagnostic accuracy based on the proposed cutoff in the validation study. Spearman correlation analysis was applied to assess the correlation of serum MMP-7 with age as well as the grade of inflammation and stage of fibrosis by liver biopsy. Random effect model was used to assess the variation of MMP-7 levels over three durations of sample frozen, as it accounts for the correlation between repeated measurements within the same sample.

No missing data were imputed to avoid introducing potential bias, and unadjusted *P* values were reported. All data analyses were performed using R software 3.6.3 (R Foundation, Vienna, Austria). A statistically significant difference was defined as a *P* value < 0.05. This work has been reported in line with the STARD (Standards for the Reporting of Diagnostic accuracy studies) criteria [26]. All authors had access to the study dataset and reviewed and approved the final manuscript.

## Results

### Diagnosis of enrolled subjects

As shown in Figs. 1 and 284 eligible infants were enrolled in Cohort A as non-affected controls. These patients were admitted to the hospital for various diagnoses, including polydactyly (*n* = 16), Hirchsprung disease (*n* = 19), imperforate anus (*n* = 17), anal fistula(*n* = 7), brachial plexus injuries (*n* = 29), inguinal hernia (*n* = 27), cleft lip or palate (*n* = 8), congenital heart disease (*n* = 16), hypertrophic pyloric stenosis (*n* = 2), neonatal omphalitis (*n* = 6), constipation (*n* = 28), pneumonia (*n* = 25), hydrocephalus (*n* = 9), convulsion or epilepsy (*n* = 17), trauma (*n* = 2), hemangioma(*n* = 9), dermatitis(*n* = 9), cryptorchidism(*n* = 6), hydronephrosis(*n* = 15), and routine body exam (*n* = 17).

Cohort B consists of 318 cholestatic infants, 172 of which were diagnosed with BA later, while 146 were confirmed cholestasis with non-BA causes. After excluding 29 patients due to refusal of further diagnostics or treatment, the prospective Cohort C consists of 687 cholestatic infants. 395 were later diagnosed with BA, and 292 were confirmed to have non-BA cholestasis (with detailed diagnoses listed in Table S1).

### Distribution of serum MMP-7 concentrations in the controls (cohort A)

**Fig. 2:**
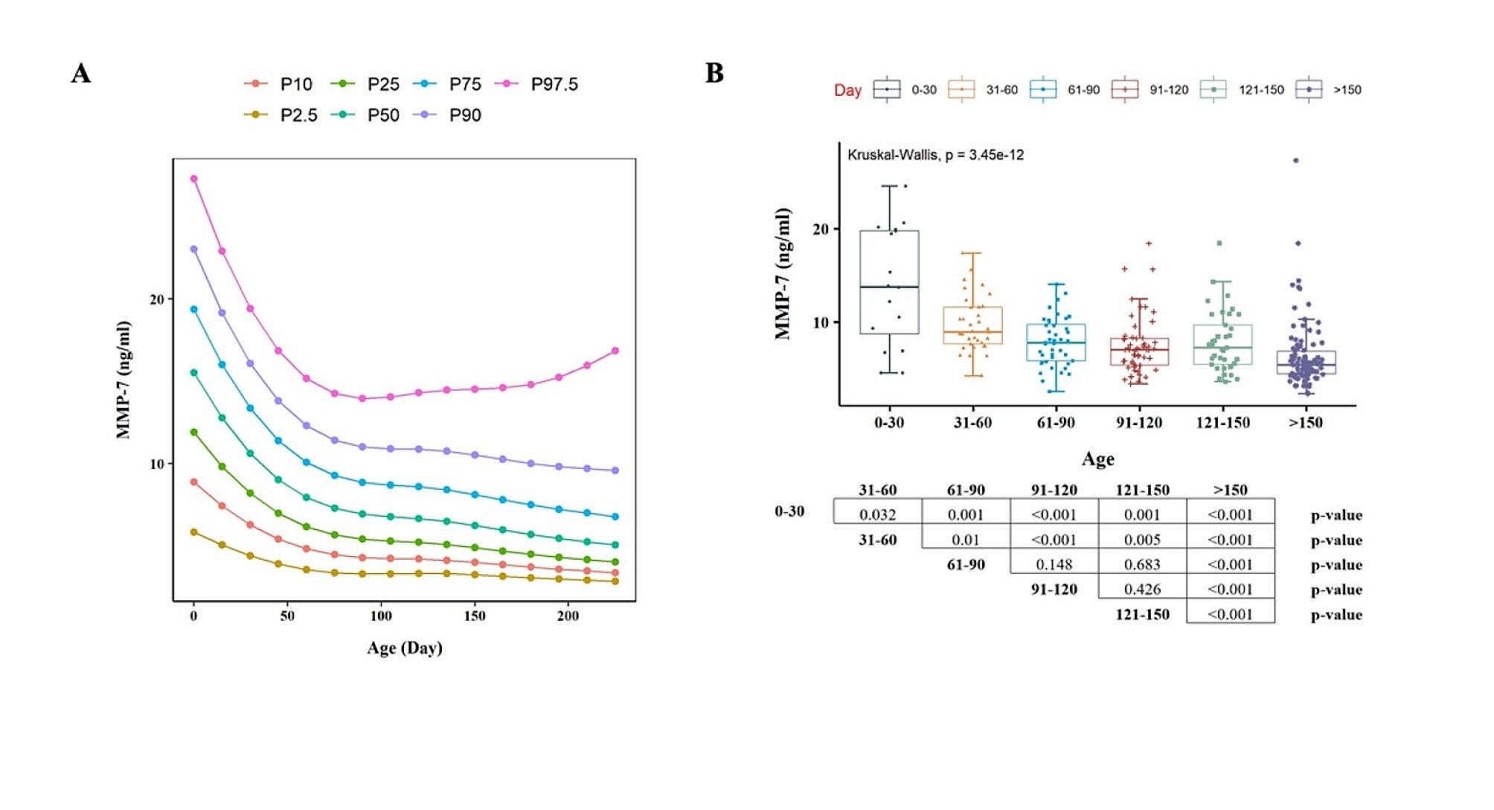
Serum MMP-7 concentration in normal controls. **(A)** Standard reference curve established using LMS method. 2.5%, 10%, 25%, 50%, 75%, 90%, 97.5% reference curves were shown. **(B)** Serum MMP-7 concentration of each age group. Boxes and whiskers represent median and interquartile range. Kruskal-Wallis tests were applied for multiple comparisons. Between-group comparisons were performed using Mann–Whitney U tests, and P values were based on bonferroni adjustment

As shown in Table 1, over half of the patients were male (58.8%). The median serum levels of TB, DB, GGT, AST, ALT, ALP, and TBA were all within the normal range. The median serum MMP-7 percentiles declined non-linearly with age (Fig. 2A), and was the highest in the group aged 0–30 d (13.76 ng/mL, interquartile range [IQR]: 8.70, 19.76). Serum MMP-7 values did not significantly differ among the five age groups (31–60 d, 61–90 d, 91–120 d, 121–150 d, and > 150 d) after Bonferroni adjustment (Fig. 2B). The 2.5th and 97.5th percentiles of MMP-7 were 4.5-23.15 ng/mL in the neonatal subgroup, and 3.34–13.98 ng/mL in infants aged older than 30 days.

**Table 1:** Demographic and clinical characteristics of study cohorts

	Cohort A Non-affected infants (*N* = 284)	Cohort B Retrospective cohort (*N* = 318)	Cohort C Prospective cohort (*N* = 687)
Sex (Male), N(%)	167 (58.8%)	187 (58.8%)	399 (58.1%)
Ethnicity (Han), N(%)	278(97.9%)	312(98.1%)	670(97.5%)
Age, days^a^	115 (71, 177)	60 (46, 76)	59 (43, 77)
GGT, IU/L^a^	23.1 (14.1, 48.0)	215.4 (113.0, 453.2)	230.6 (121.8, 477.4)
AST, IU/L^a^	40.7 (33.2, 51.4)	196.3 (125.5, 294.9)	191.8 (122.4, 314.7)
ALT, IU/L^a^	25.4 (19.6, 35.5)	127.6 (71.0, 202.9)	130.3 (75.7, 226.5)
TB, umol/L^a^	6.2 (4.1, 10.1)	168.1 (131.8, 209.3)	141.1 (107.9, 179.4)
DB, umol/L^a^	2.5 (1.7, 4.3)	107.2 (82.6, 137.6)	106.4 (81.2, 134.1)
TBA, umol/L^a^	6.8 (4.7, 11.3)	107.6 (84.8, 134.9)	96.1 (72.0, 124.4)
with CHD, N(%)	/	26(8.2%)	67(9.8%)
Inflammation stages, N G1/G2/G3/G4	/	8/102/128/1	2/109/346/2
Fibrosis stages, N S0/S1/S2/S3/S4	/	2/38/118/70/12	3/20/193/212/31

^a^: Values are median (Q1, Q3)

BA: biliary atresia; CHD: congenital heart disease; GGT: gamma glutamyl transferase; AST: aspartate aminotransferase; ALT: alanine aminotransferase; TB: total bilirubin; DB: direct bilirubin; TBA: total bile acid

### The cutoff value and diagnostic performance of serum MMP-7 in cholestatic patients (cohort B)

**Fig. 3:**
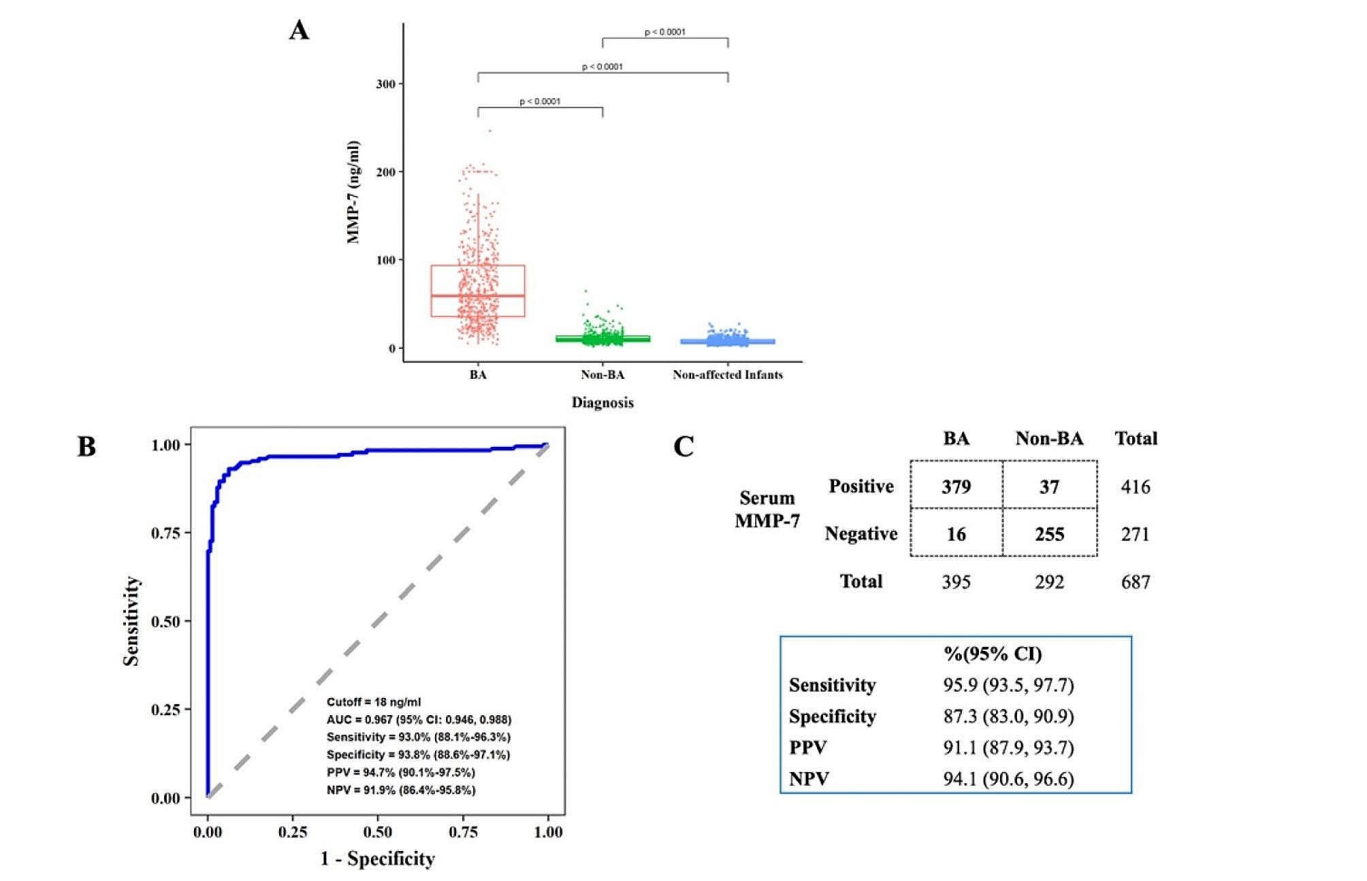
Diagnostic accuracy of serum MMP-7 in cholestatic patients. **(A)** Serum MMP-7 concentration in BA, non-BA and normal control groups. Boxes and whiskers represent median and interquartile range. Between-group comparisons were performed using Mann–Whitney U tests. **(B)** Receiver operating characteristic (ROC) plots based on the retrospective cohort **(C)** “2 × 2” table in the validation prospective cohort based on the cutoff value proposed from retrospective cohort (18ng/ml)

As shown in Table 1 and Table S2, the three cohorts and also BA and non-BA groups had similar age, gender and ethnicity proportions. There were significant differences between BA and non-BA groups in GGT and DB. Serum MMP-7 was similar between male and female patients (Table S3), but significantly differed among BA, non-BA patients and normal controls (Fig. 3A). In the retrospective cohort, the median serum MMP-7 level was higher in the BA group than that in the non-BA group (56.87 ng/mL (IQR: 32.64, 89.42) vs. 9.40 ng/mL (IQR: 7.30, 12.00); *P* < 0.001). An AUC value of 0.967 (95% CI: 0.947–0.988) was obtained for serum MMP-7. A cutoff value of 18 ng/mL achieved a sensitivity, specificity, PPV, and NPV of 93.0% (95% CI: 88.1–96.3%), 93.8% (95% CI: 88.6–97.1%), 94.7% (95% CI: 90.1–97.5%), and 91.9% (95% CI: 86.4–95.8%), respectively (Fig. 3B).

### Prospective validation of serum MMP-7 in cholestatic patients (cohort C)

The cutoff value of 18 ng/mL of serum MMP-7 levels classified 395 patients as BA and 292 as non-BA cases in the prospective cohort, resulting in a diagnostic sensitivity of 95.9% (379/395; 95% CI: 93.5–97.7%), a specificity of 87.3% (255/292; 95% CI: 83.0–90.9%), a PPV of 91.1% (379/416; 95% CI: 87.9–93.7%), and a NPV of 94.1% (255/271; 95% CI: 90.6–96.6%), respectively (Fig. 3C). The 37 infants with false positive test of MMP-7 were further diagnosed as idiopathic cholestasis (*n* = 22), CMV hepatitis (*n* = 1), choledochal cyst (*n* = 3), Alagille syndrome (*n* = 3), citrin deficiency (*n* = 1), PFIC (*n* = 1), parenteral nutrition-associated cholestasis (*n* = 3), inborn errors of bile acid synthesis (*n* = 2) and bile duct dysplasia (*n* = 1). All infants with false negative results (*n* = 16) underwent intraoperative cholangiography and were subsequently diagnosed with BA and underwent KPE by the same surgeon (SZ).

### Diagnostic performance of serum MMP-7 in each age group for cholestatic patients

**Fig. 4:**
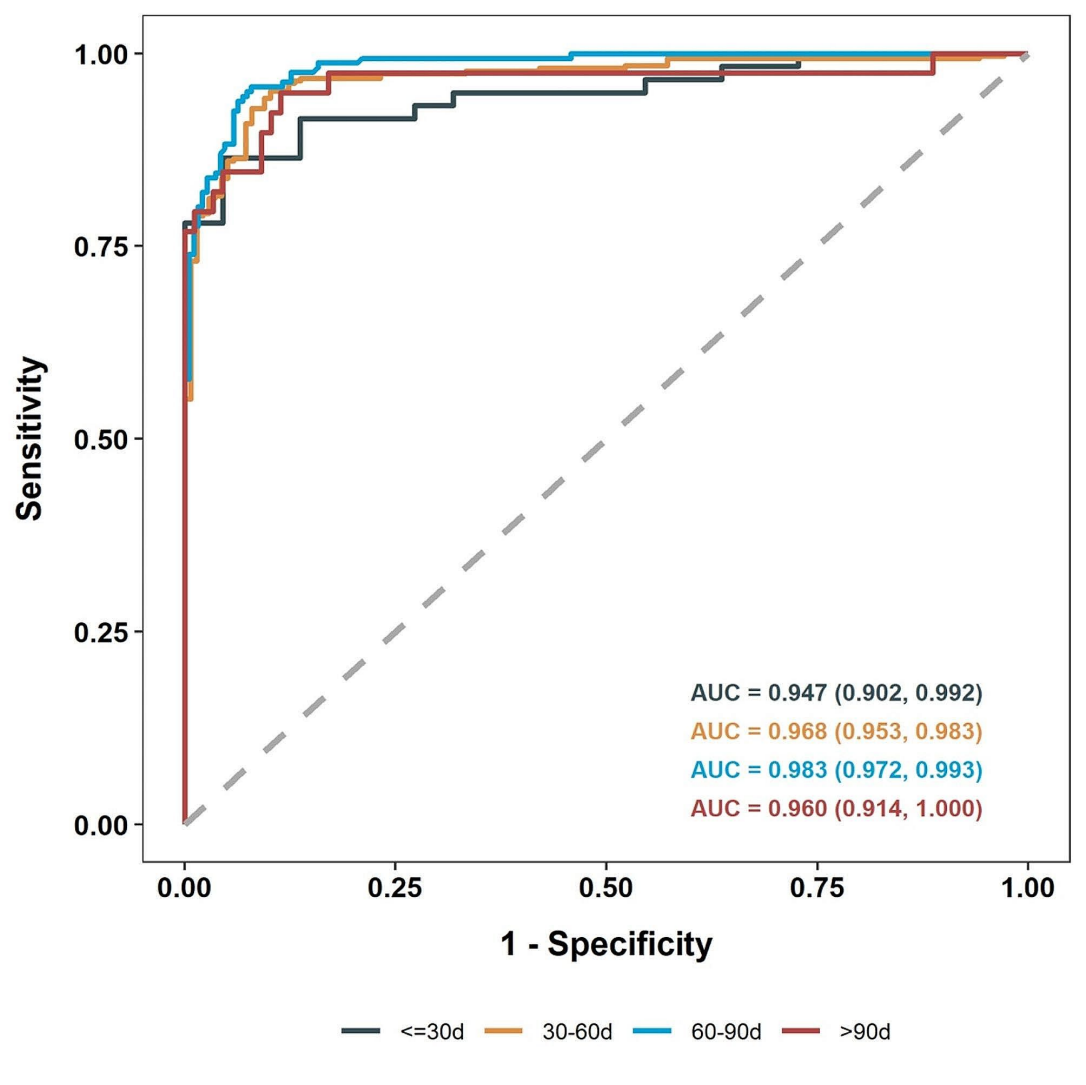
Diagnostic accuracy of serum MMP-7 in cholestatic patients for each age group. Receiver operating characteristic (ROC) plots for each age group. (≤ 30 d: AUC = 0.947(95%CI: 0.902,0.992), 30–60 d: AUC = 0.968(95%CI: 0.953,0.983), 60–90 d: AUC = 0.983(95%CI: 0.972,0.993), > 90 d: AUC = 0.96(95%CI: 0.914,1)

Given that the distribution of serum MMP-7 with age varied between BA and non-BA patients (Figure S1), the diagnostic accuracy of MMP-7 was further tested by age groups: 0–30 d, 31–60 d, 61–90 d, and > 90 d. The ideal diagnostic performance of MMP-7 cutoff of 18 ng/ml remained but not achieved in the 0–30 d group, including 59 BA and 22 non-BA infants, yielding a sensitivity, specificity, PPV, and NPV of 93.2% (95% CI: 83.5–98.1%), 73.7% (95% CI: 49.8–89.3%), 90.2% (95% CI: 79.8–96.3%), and 80% (95% CI: 56.3–94.3%), respectively. The median serum MMP-7 level were higher in the 0–30 d group, especially in the non-BA infants compared with other age groups (51.30 ng/mL (IQR: 38.66, 87.66) for BA vs. 15.75 ng/mL (IQR: 10.12, 18.97) for non-BA; *P* < 0.001). Serum MMP-7 achieved an AUC value of 0.947 (95% CI: 0.902–0.992) in differentiating BA in neonates aged 0–30 d, with a cutoff value of 28.1 ng/mL achieving the best diagnostic performance for this group, a sensitivity, specificity, PPV, and NPV of 86.4% (95% CI: 75.0–94.0%), 95.5% (95% CI: 77.2–99.9%), 98.1% (95% CI: 89.7–100%), and 72.4% (95% CI: 52.8–87.3%), respectively (Fig. 4; Table 2).

**Table 2:** Diagnostic accuracy of serum MMP-7 by age group

Age Group	*N*	AUC (95%CI)	Cut-off value (ng/ml)		BA	Non-BA	Sensitivity (%) (95%CI)	Specificity (%) (95%CI)	PPV (%) (95%CI)	NPV (%) (95%CI)
0-30d	81	0.947 (0.902, 0.992)	28.1	+	51	1	86.4 (75.0–94.0)	95.5 (77.2–99.9)	98.1 (89.7–100.0)	72.4 (52.8–87.3)
				-	8	21				
			18	+	55	6	93.2 (83.5–98.1)	72.7 (49.8–89.3)	90.2 (79.8–96.3)	80.0 (56.3–94.3)
				-	4	16				
31-60d	446	0.968 (0.953, 0.983)	18	+	293	15	95.1 (92.1–97.2)	89.1 (82.7–93.8)	95.1 (92.1–97.2)	89.1 (82.7–93.8)
				-	15	124				
61-90d	351	0.983 (0.972, 0.993)	18	+	154	15	95.7 (91.2–98.2)	92.1 (87.3–95.5)	91.1 (85.8–94.9)	96.2 (92.2–98.4)
				-	7	175				
> 90d	127	0.960 (0.914, 1.000)	18	+	37	10	94.9 (82.7–99.4)	88.6 (80.1–94.4)	78.7 (64.3–89.3)	97.5 (91.3–99.7)
				-	2	78				

AUC: the area under the ROC curve; BA: biliary atresia; CI: confidence interval; PPV: positive predictive value; NPV: negative predictive value

### Correlation of serum MMP-7 with inflammation grade and fibrosis stage of liver biopsy

Liver biopsy was available for 699 infants, 526 of which were BA patients and 173 were non-BA patients. MMP-7 showed a weak significant correlation with the fibrosis stage in the BA group (*R* = 0.48, *P* < 0.001), as well as a weak significant correlation with the inflammation grade (*R* = 0.17, *P* < 0.001), whereas in non-BA group, MMP-7 showed a weak significant correlation with the inflammation grade (*R* = 0.24, *P* = 0.004), but no significant correlation with the fibrosis stage(*R* = 0.29, *P* = 0.056) (Figure S2).

## Discussion

Our study with a large sample size of its kind and an independent prospective cohort for validation demonstrates a non-linear centile distribution of serum MMP-7 with age in non-affected controls and provides stronger evidence supporting the diagnostic accuracy of serum MMP-7 as a biomarker for discriminating BA from other pediatric cholestatic patients. We proposes a set of age-specific cutoffs with higher cutoff value of 28 ng/ml for neonatal patients to achieve best discrimination, further improving early BA diagnosis, laying the foundation for its clinical application and popularization in the diagnostic and screening algorithm for BA.

We found that the serum MMP-7 levels declined non-linearly with age in the non-affected infant population, which has not been reported previously. Bias might be introduced because a wide range of non-hepatobiliary conditions were included. There is possibility that some disease conditions might alter the MMP-7 levels. However, the range of MMP-7 levels was narrow in each age group, and the large sample size and variety of diseases further generalized our results. The 2.5th and 97.5th percentiles of MMP-7 concentration were 4.5-23.06 ng/mL in the neonatal subgroup (aged 0–30 days), and 3.13–15.57 ng/mL in infants aged 30 days and over. MMPs are involved in ECM remodeling, which is closely related to various physical and pathological processes, including fetal development, tissue regeneration, and fibrosis [27, 28]. This might explain higher serum MMP-7 levels in neonates of the healthy infant group who grew rapidly and whose MMP-7 levels vary largely accordingly. Such trend reveals that neonatal patients with higher MMP-7 levels may need a higher cutoff value.

We identified a cutoff value of 18 ng/ml with good performance for identifying BA from all pediatric cholestatic patients, which was validated prospectively in a larger cohort. Besides, we proposed a higher MMP-7 cutoff of 28.1 ng/mL for the neonatal patients to achieve best discrimination which was in line with its age distribution. The diagnostic accuracy was a little reduced in neonates, hard to maintain both sensitivity and specificity around 90%. Compared with 18 ng/ml, the cutoff of 28.1 ng/ml demonstrated higher specificity in neonates, which is important to avoid unnecessary invasive diagnostic surgery. The cutoff of 18 ng/ml demonstrated higher sensitivity in neonates, indicating these patients should still be closely followed up. The age-specific cutoff is of great value in accurate and early diagnosis of BA, critical for timely surgical intervention and prognosis improvement.

Serum MMP-7 holds great promise for diagnosing BA with AUCs all > 0.9 [14–16, 18, 20–24], superior to other diagnostic methods including GGT and ultrasound, however, optimal cutoff values proposed varied significantly across these studies (52.85 ng/mL by Yang et al. [14]. , 10.37 ng/mL by Jiang et al. [16]. , 1.43 ng/mL by Wu et al. [15]. , 18.6 ng/ml by Sakaguchi et al. [21]. , 7.8 ng/ml by Rohani et al. [20]. , 4.99 ng/ml by Singh et al. [22]. , 26.7 ng/ml by Chi et al. [18]. , and 69 ng/ml by Aldeiri et al. [19] These inconclusive cutoffs limited the clinical applications of this biomarker. Compared with our previous study [16], the serum MMP-7 levels were higher, which may be explained by laboratory assay issues. We used a different ELISA kit, which proved to be more stable, and the blood samples used for these assays were several months for the retrospective cohort and no more than 7 days for the prospective cohort, respectively. Stability tests of serum samples in this study indicated that it is stable to measure MMP-7 within at least 7 months, with variation over time below 15% (Table S4). A cutoff value of 1.43 ng/mL was proposed by Wu et al. using another kit (ELISA; DuoSet, R&D Systems, Inc, Minneapolis, Minnesota) from the same company, however, the serum samples were stored for up to 10 years before examination [15]. A degree of protein degradation may happen over decades. This may partly explain the difference in MMP-7 levels across studies. Another study by Aldeiri et al. [19]also used stored serum samples with a median storage time of 12 years, but yielded a much higher cutoff value of 69 ng/ml. The kit used was different (Human MMP-7/Matrilysin ELISA Kit PicoKine, Cat#EK0463, Boster, Boster Biological Technology, Pleasanton CA, USA). A more recent study from North America adopted another two different assays including Millipore^®^ Luminex assay and Time Resolved-Fluorescence Energy Transfer (TR-FRET) and proposed two different cutoffs (52.8ng/ml for Luminex and 18.2ng/ml for TR-FRET). Thus, we believe methodological differences, such as serum sample storage conditions and interval, kits selection and experimental procedures, may explain the huge variation in studies published so far. Fresh sample and unified kit selection are highly recommended for further standardization of MMP-7 measurement.

In the prospective cohort, MMP-7 misclassified 37 and 16 infants with false-positive and false-negative results, respectively. MMP-7 is secreted from cholangiocytes and hepatic stellate cells following liver injury or fibrosis, and serum MMP-7 levels correlated with fibrosis stage on liver biopsy [16]. Patients with other hepatobiliary diseases can also have liver fibrosis and elevated MMP-7 levels, however, around the cutoff value, indicating that the regulatory pathways of MMP-7 in non-BA may be different from those associated with BA. Diagnostic surgery was recommended for all false negative patients because of their clinical symptoms and other biochemistry results. Therefore, based on our clinical application of MMP-7, there is no overlap in the median MMP-7 levels between BA and non-BA patients, so high MMP-7 values (far beyond the cutoff) are all consistent with the diagnosis of BA. However, even if the MMP-7 level is below the cutoff, the infant should be closely followed up until the jaundice clears or the suspicion for BA is totally eliminated. Otherwise, diagnostic surgery should be performed as soon as possible. Likewise, if the MMP-7 is around cutoff, diagnostic surgery could be postponed if other clinical symptoms and biochemistry results do not support the diagnosis of BA.

The mechanism through which MMP-7 affects the pathophysiology of BA remains unclear. In general, as reported previously, MMP-7 levels correlate with liver fibrosis in BA patients [12], which was also observed in our study, while MMP-7 showed no significant correlation with the fibrosis stage in non-BA cholestasis, indicating MMP-7 might be involved in a specific pathway of liver fibrosis for BA. Additionally, some studies demonstrated that elevated MMP-7 levels post-KPE were observed in patients with poor outcomes [15, 18, 29], while Sakaguchi et al. found MMP-7 could not predict liver transplantation within a year [21]. Whether there is a correlation between high pre-KPE MMP-7 levels and post-KPE outcomes as well as dynamic trend of MMP-7 post-KPE warrants longitudinal investigation in the future, which might be helpful in predicting the prognosis and determining the time of liver transplantation. However, the 16 false negative patients with pre-Kasai MMP-7 levels below 18 ng/ml in the prospective cohort are found with poor outcomes after the Kasai procedure. The correlation between low MMP-7 levels even below the cutoff value pre-KPE and poor outcomes is of great interest. Future studies with larger sample sizes and long-term follow-up are warranted to determine its potential as a new BA classification that could contribute to designing new treatment strategies for BA in the future, like direct liver transplantation instead of palliative surgery. We are continuously following up on such patients and collecting data for testing this hypothesis.

However, our study inevitably has limitations. First, given the large sample size and independent external validation with about 70% patients being non-local, this is still a single-center study, which limits the generalizability. Multi-center studies are warranted to further validate our findings, especially the diagnostic accuracy in neonates, which is of great significance in earlier identification of BA and improving prognosis. Second, serum MMP-7 levels may vary due to external factors besides age, such as nutrition, concurrent infections, or genetic predispositions. Also, MMP-7 have been studied in the pathogenesis of various disease processes including pulmonary and renal fibrosis, inflammatory bowel disease, and carcinogenesis [28]. However, we were unable to investigate all these potential confounders in this study set. Continued research into external influencing factors on serum MMP-7 levels as well as the mechanistic role of MMP-7 in BA is advocated. Additionally, some studies have investigated the value of combining MMP-7 with other biomarkers of liver function or bile acid, but the accuracy remained similar [14, 16, 24]. The diagnostic accuracy of multi-variate diagnostic model combined with MMP-7 and other parameters like ultrasound may be worth investigating. Last, some diagnoses, such as alpha-1-antitrypsin deficiency which might mimic BA but is rare in Chinese infants, were not included in the non-BA group, which may limit the generalizability in other racial populations.

The diagnostic accuracy of MMP-7 for BA was widely approved. Selection of ELISA kits, sample storage conditions and experimental procedures should be considered for standard MMP-7 measurement before its intergradation into clinical diagnostic algorithm. Continued research into predictive value as well as the mechanistic role of MMP-7 in BA is advocated.

## Conclusions

The study conclusively demonstrates the diagnostic value of serum MMP-7 as a biomarker for biliary atresia in the Chinese pediatric population, establishing age-specific cutoff values for improved accuracy. This includes a novel, higher cutoff for neonates, addressing a critical gap in early BA diagnosis. These findings hold the potential to significantly enhance clinical decision-making, particularly in early and accurate BA identification, thereby improving patient outcomes. However, the study’s limitations highlight the need for further validation across diverse populations and multicenter settings. We advocate for continued research into the mechanistic role of MMP-7 in BA and its integration into clinical practice, underscoring the importance of standardized approaches in biomarker measurement and application.

### Electronic supplementary material

Below is the link to the electronic supplementary material.

Supplementary Material 1

Supplementary Material 2

## Data Availability

The data that support the findings of this study are available from the corresponding author upon reasonable request.
