# Supplementary material for: Serum matrix metalloproteinase-7 for discriminating biliary atresia: a diagnostic accuracy and validation study
Source: J Transl Med. 2024 Jul 8;22:636. doi: 10.1186/s12967-024-05442-x (PMC11229253; doi:10.1186/s12967-024-05442-x)

**Contents**

| **Table S1. Final diagnosis of non-BA cholestatic subjects in Cohort B and Cohort C** | **Page 2** |
| --- | --- |
| **Table S2. Demographic and clinical characteristics of BA vs Non-BA patients in Cohort B and Cohort C** | **Page 3** |
| **Table S3. Serum MMP-7 levels in male and female cholestatic subjects** | **Page 4** |
| **Table S4. Long-term stability of the serum samples stored at -80℃** | **Page 5** |
| **Figure S1. Correlation of serum MMP-7 and age in cholestatic patients** | **Page 6** |
| **Figure S2. Correlation of serum MMP-7 and inflammation grade and fibrosis stage of liver biopsy** | **Page 7** |

**Table S1. Final diagnosis of non-BA cholestatic subjects in Cohort B and Cohort C**

| **Diagnosis** | **Cohort B**  **Retrospective cohort**  **(N=146)** | **Cohort C**  **Prospective cohort**  **(N=292)** |
| --- | --- | --- |
| CMV infection | 16 | 19 |
| PN related cholestasis | 4 | 35 |
| Alagille syndrome | 8 | 21 |
| NICCD | 7 | 15 |
| PFIC | 4 | 14 |
| Dubin-Johnson syndrome | 1 | 8 |
| Intrahepatic bile duct dysplasia | 1 | 1 |
| Choledocal cyst | 0 | 10 |
| Niemann-Pick disease | 1 | 1 |
| Noonan syndrome | 0 | 2 |
| inborn errors of bile acid synthesis | 0 | 17 |
| Idiopathic cholestasis | 104 | 149 |

CMV: Cytomegalovirus; PN: Parenteral nutrition; NICCD: Neonatal intrahepatic cholestasis caused by citrin deficiency; PFIC: Progressive familial intrahepatic cholestasis

**Table S2. Demographic and clinical characteristics of BA vs Non-BA patients in Cohort B and Cohort C**

|  | **Retrospective Cohort (N=318)** | | **P value*** | **Prospective Cohort (N=687)** | | **P value*** |
| --- | --- | --- | --- | --- | --- | --- |
|  | **BA (N=172)** | **Non-BA (N=146)** |  | **BA (N=395)** | **Non-BA (N=292)** |  |
| Gender (Male), N(%) | 90 (52.3%) | 97 (66.4%) | 0.015 | 195 (49.5%) | 203 (69.5%) | <0.001 |
| Age, days^a^ | 56 (43, 66) | 65 (53, 85) | <0.001 | 53 (39, 68) | 70 (50, 86) | <0.001 |
| GGT, IU/L^a^ | 399.8 (209.9, 779.4) | 119.5 (74.3, 210.9) | <0.001 | 399.2 (203.8, 637.2) | 115.4 (66.8, 185.0) | <0.001 |
| AST, IU/L^a^ | 193.8 (129.3, 277.8) | 200.9 (117.5, 383.5) | 0.548 | 193.5 (124.5, 305.7) | 188.5 (117.2, 329.1) | 0.667 |
| ALT, IU/L^a^ | 127.6 (70.8, 179.2) | 126.8 (72.0, 271.2) | 0.210 | 122.6 (78.0, 203.8) | 137.8 (73.9, 273.5) | 0.046 |
| TB, umol/L^a^ | 168.3 (137.0, 206.8) | 165.7 (120.8, 221.4) | 0.451 | 148.6 (121.1, 182.2) | 125.0 (90.2, 175.7) | <0.001 |
| DB, umol/L^a^ | 116.2 (92.9, 139.8) | 93.6 (73.9, 127.2) | <0.001 | 113.7 (91.4, 137.1) | 93.4 (66.8, 127.8) | <0.001 |
| TBA, umol/L^a^ | 105.5 (86.5, 129.2) | 113.7 (77.9, 141.6) | 0.487 | 98.9 (75.9, 125.8) | 90.1 (61.8, 121.6) | 0.005 |
| with CHD, N(%) | 26(15.1%) | / | / | 67(17.0%) | / | / |
| Inflammation stages, N  G1/G2/G3/G4 | 1/64/101/1 | 7/38/27/0 | <0.001 | 1/76/280/2 | 1/33/66/0 | <0.001 |
| Fibrosis stages, N  S0/S1/S2/S3/S4 | 0/2/92/61/12 | 2/36/26/9/0 | <0.01 | 0/3/138/188/30 | 3/17/55/24/1 | <0.001 |

^a^: Values are median (Q1, Q3)

^*^: P value between BA and Non-BA groups. Chi-square tests were applied to gender, inflammation stages and fibrosis stages, while the rest were tested by Mann-Whitney test.

BA: biliary atresia; CHD: congenital heart disease; GGT: gamma glutamyl transferase; AST: aspartate aminotransferase; ALT: alanine aminotransferase; TB: total bilirubin; DB: direct bilirubin; TBA: total bile acid

**Table S3. Serum MMP-7 levels in male and female cholestatic subjects**

|  | **Serum MMP-7 levels** ^a^ | | | | |
| --- | --- | --- | --- | --- | --- |
|  | **Male** | | **Female** | | **P value**^*^ |
| **Cohort B (N=318)** |  |  |  |  |  |
| **BA** | **N=90** | 56.24 (36.56, 87.56) | **N=82** | 57.67 (30.85, 92.40) | 0.643 |
| **Non-BA** | **N=97** | 9.61 (7.30, 12.73) | **N=49** | 8.71 (7.31, 11.20) | 0.316 |
| **Cohort C (N=687)** |  |  |  |  |  |
| **BA** | **N=196** | 58.26 (34.83, 88.63) | **N=199** | 62.91 (38.66, 97.58) | 0.154 |
| **Non-BA** | **N=203** | 10.50 (7.45, 14.34) | **N=89** | 10.67 (7.61, 14.01) | 0.943 |

^a^: median (Q1, Q3)

^*:^ P value between male and female groups. Mann-Whitney tests were applied.

BA: biliary atresia; MMP-7: matrix metalloproteinase 7

**Table S4. Long-term stability of the serum samples stored at -80℃**

| **Sample** | **Fresh** | **4 Months** | | **7 Months** | |
| --- | --- | --- | --- | --- | --- |
|  | **MMP-7 levels(ng/ml)** | **MMP-7 levels(ng/ml)** | **Deviation^*^** | **MMP-7 levels(ng/ml)** | **Deviation^*^** |
| **Sample 1** | 8.59 | 9.31 | 8.3% | 7.78 | -9.5% |
| **Sample 2** | 53.66 | 56.61 | 5.5% | 52.03 | -3.0% |
| **Sample 3** | 16.65 | 14.00 | -15.9% | 15.30 | -8.1% |
| **Sample 4** | 62.04 | 56.70 | -8.6% | 61.70 | -0.5% |
| **Sample 5** | 8.98 | 7.59 | -15.4% | 9.76 | 8.7% |
| **Sample 6** | 9.37 | 8.38 | -10.6% | 9.41 | 0.4% |
| **Sample 7** | 8.59 | 8.49 | -1.1% | 7.44 | -13.4% |
| **Sample 8** | 9.89 | 11.03 | 11.6% | 8.46 | -14.4% |
| **Sample 9** | 17.29 | 16.95 | -2.0% | 15.85 | -8.3% |
| **Sample 10** | 42.87 | 50.72 | 18.3% | 40.01 | -6.7% |
| **Sample 11** | 74.11 | 72.09 | -2.7% | 69.70 | -5.9% |
| **Sample 12** | 49.52 | 57.39 | 15.9% | 51.30 | 3.6% |

^*^: Fresh MMP-7 level was used as the reference for deviation calculation.

Generalized equation estimation model for continuous response variable was used to test the significance of MMP-7 levels from fresh serum sample (as baseline) and that from serum stored fro 4 months and 7 months. Time was coded as 0, 1 and 2 respectively and treated as categorical variables in the regression model. Family function of Gaussian and link function of identity was used, based on assumption of exchangeable correlation structure. All MMP-7 raw data were log-transformed before analysis to obtain approximate normal distribution. Model P =0.184, indicating a non-statistical variation of MMP-7 over time.

**Figure S1. Correlation of serum MMP-7 and age in cholestatic patients.** Boxes and whiskers represent median and interquartile range. **(A)** Linear regression curve of serum MMP-7 and age **(B)** For BA group **(C)** For non-BA group


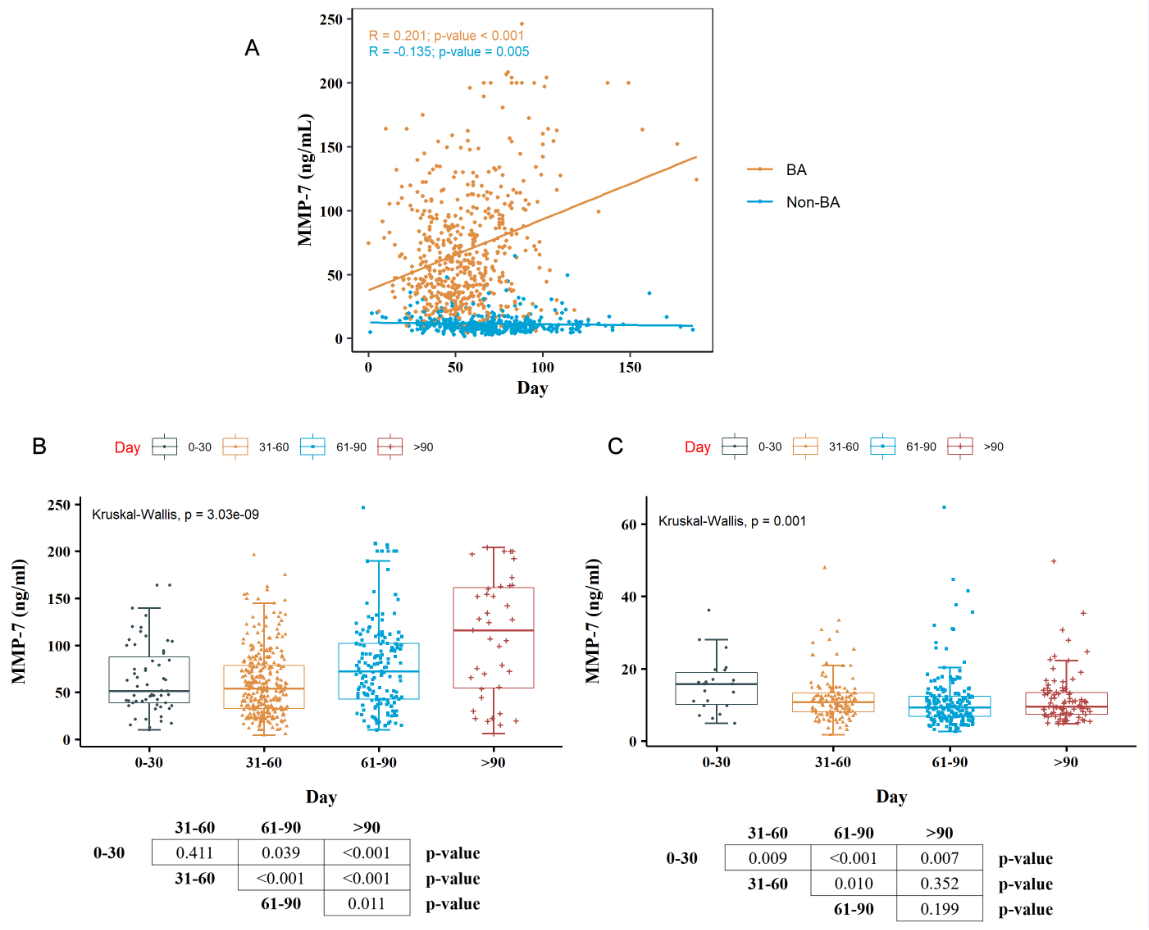


**Figure S2. Correlation of serum MMP-7 and inflammation grade and fibrosis stage of liver biopsy.** Boxes and whiskers represent median and interquartile range. **(A)** For all cholestatic subjects **(B)** For BA group **(C)** For non-BA group


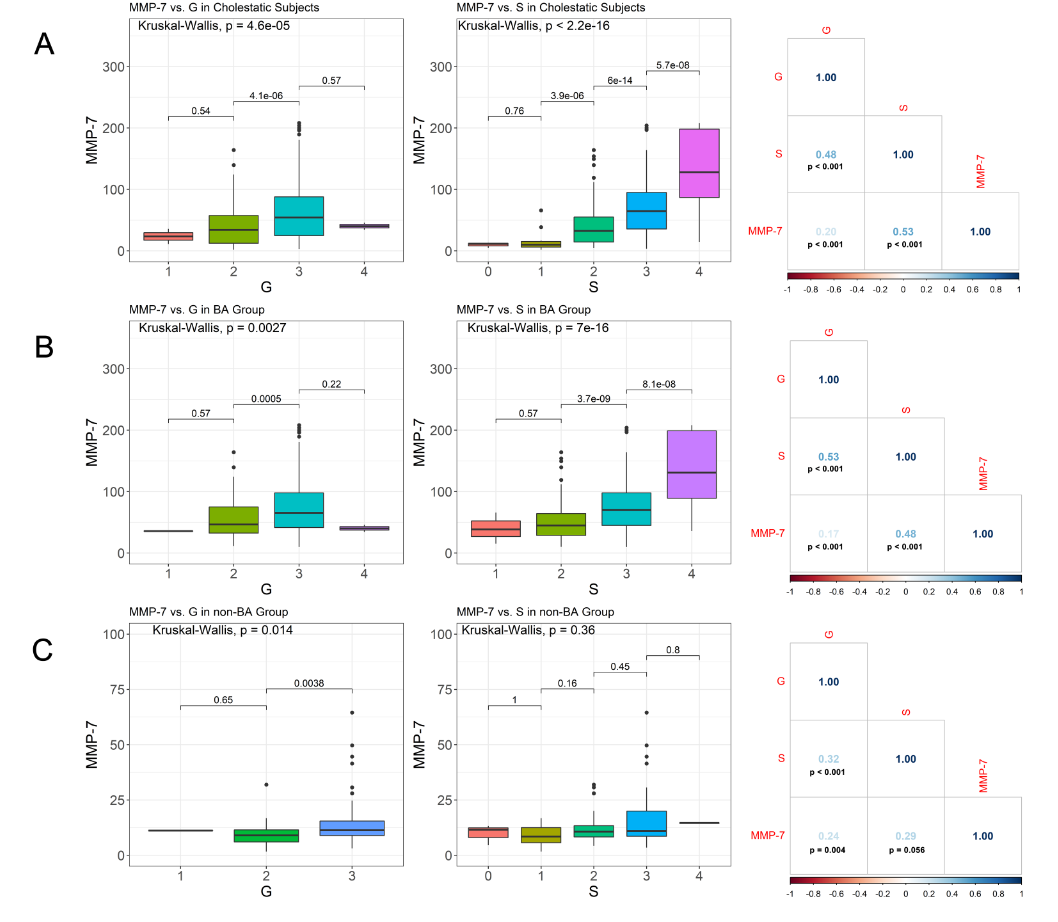

Supplement: Supplementary file 2 — Supplementary Material 2 [file 12967_2024_5442_MOESM2_ESM.docx]
